# Macrophage USF2 Lactylation‐Mediated FABP4 Suppression Promotes Intracellular Proliferation of *Brucella*

**DOI:** 10.1155/tbed/3930677

**Published:** 2026-11-04

**Authors:** Yuanhao Yang, Yaping Zu, Xin Wang, Xiaofang Liu, Yunyi Zhai, Ye Yuan, Gaowa Wudong, Wei Liu, Yaping Jin, Dong Zhou, Aihua Wang

**Affiliations:** ^1^ College of Veterinary Medicine Northwest A&F University Yangling 712100 Shaanxi China nwsuaf.edu.cn; ^2^ Key Laboratory of Animal Biotechnology of the Ministry of Agriculture Northwest A&F University Yangling 712100 Shaanxi China nwsuaf.edu.cn

**Keywords:** *Brucella abortus* A19, Fabp4, lactylation, SepA, Slc2a1, USF2

## Abstract

*Brucella* spp. are facultative intracellular bacteria that infect and induce brucellosis in a diverse range of mammalian hosts. Understanding the mechanisms underlying the intracellular survival of *Brucella* is of critical importance for the prevention and control of brucellosis. This study uncovers the mechanism by which *Brucella abortus* manipulates host lactate metabolism to facilitate its intracellular proliferation. Infection of macrophages by *B. abortus* enhanced L‐lactate production by the host macrophages, which in turn promoted bacterial proliferation. Proteomic and functional analyses identified the host glucose transporter protein Slc2a1 as a positive regulator of both *B. abortus* proliferation and host cellular lactylation. The *B. abortus* effector protein SepA upregulated Slc2a1 expression and host cellular lactylation. Lactylation of the transcription factor USF2 at lysine residues 255 and 275 suppressed production of the host lipid metabolism‐associated protein Fabp4, thereby enhancing bacterial survival. Moreover, Fabp4 inhibition attenuated *B. abortus*‐induced M1 polarization and lipid metabolism in macrophages. These findings reveal a dual‐axis regulatory network through which *B. abortus* exploits host L‐lactate metabolic reprogramming via the SepA/Slc2a1 and L‐lactate‐USF2/Fabp4 axes to establish its intracellular niche, providing new insights into host–*Brucella* metabolic crosstalk.

## 1. Introduction

Brucellosis is a zoonotic infectious disease. In humans, brucellosis infection presents with acute febrile illness and chronic infection and may be associated with a spectrum of nonspecific clinical manifestations, including fatigue, headache, diaphoresis, lassitude, depression, anorexia, abdominal pain, and back pain [1]. The bacteria that cause brucellosis belong to the genus *Brucella* and are facultative intracellular pathogens. The genus *Brucella* comprises 12 species, among which three—*Brucella melitensis*, *Brucella abortus*, and *Brucella suis*—are the main pathogenic species of *Brucella* for both animals and humans [2]. *Brucella* spp. are distributed in more than 170 countries. They cause substantial economic losses by reducing livestock productivity. They also pose a major global public health threat because of their high zoonotic transmission risk, the chronic and debilitating nature of human infections, and persistent challenges in surveillance and control [3]. Infection with *Brucella*, a facultative intracellular pathogen, elicits a cascade of physiological and biochemical perturbations in host cells, accompanied by marked metabolic reprogramming. Substantial alterations in cellular metabolic homeostasis particularly involve critical pathways, including glycolysis [4–8], NAD metabolism [9], and iron regulatory networks [10]. To replicate within permissive host cell niches, intracellular bacterial pathogens must overcome hostile intracellular conditions and acquire nutrients from host cells. They achieve this by modulating host metabolic processes [11–14], thereby enhancing nutrient availability and creating a secure microenvironment that promotes bacterial proliferation.

*Brucella* infection significantly upregulates glycolysis in macrophages, leading to increased production of glycolytic end products, including lactate [4, 8]. Lactate is often mistakenly considered to be a metabolite produced only under anaerobic conditions with multiple deleterious effects [15]. However, increasing evidence indicates that lactate is an important signaling molecule rather than simply a waste product of anaerobic metabolism [16, 17]. Lactate signals through the G‐protein‐coupled receptor 81 (GPR81) or is internalized via monocarboxylate transporters [18, 19]. It is evident that lactate assumes a pivotal role in the intricate molecular crosstalk between pathogenic microorganisms and host cellular defenses during infection [20–25]. For example, lactate exerts a pivotal regulatory role in histone lactylation modifications [26]. Lactylation is a posttranslational modification process characterized by the enzymatic covalent conjugation of lactate molecules to ε‐amino groups of lysine residues within target proteins. The reaction is mediated by acyltransferase activity that modulates protein functionality through metabolic‐epigenetic crosstalk. Hypoxic conditions, stimulation with interferon‐γ, lipopolysaccharide, and bacterial infection induce histone lactylation modifications, which lead to histone accumulation at gene promoter regions, thereby regulating transcriptional activity [27]. Lactylation also extends beyond histones to nonhistone proteins, broadening its epigenetic and posttranslational regulatory scope [28–33]. Lactate functions as a signaling molecule that modulates glycolytic and lipid metabolism across diverse cell types through lactylation‐driven posttranslational modifications [27]. Lactate also contributes to immune response regulation by upregulating methyltransferase‐like 3, which facilitates immunosuppressive adaptation [34]. Additionally, lactate mediates macrophage polarization dynamics via lactylation‐mediated structural modification of pyruvate kinase M2, a critical glycolytic enzyme involved in immunometabolic reprogramming [35]. Thus, L‐lactate has transitioned from a catabolic byproduct to a multifunctional signaling mediator that contributes to epigenetic regulation through posttranslational modifications. This shift reflects a broader reappraisal of metabolic reprogramming and intercellular communication. *Brucella*, a facultative intracellular pathogen, induces dramatic metabolic reprogramming in host cells, leading to markedly increased lactate production through enhanced glycolytic flux [4, 5, 8]. Nevertheless, the role of lactate in *Brucella*‐infected host cells and whether host proteins undergo lactylation during infection remain unclear. Therefore, this study was designed to investigate these critical questions using a range of experimental approaches.

In this study, we demonstrate that *B. abortus* upregulates the expression of the host glucose transporter protein Slc2a1 via the effector protein SepA, thereby promoting L‐lactate synthesis by infected cells. This process stimulates lysine lactylation of the host USF2 transcription factor, which subsequently suppresses expression of the host lipid metabolism‐associated FABP4. Consequently, *B. abortus*‐induced M1 polarization of macrophages and lipid metabolism are inhibited, which ultimately facilitates the intracellular proliferation of the pathogen in host cells.

## 2. Materials and Methods

### 2.1. Bacterial Strains and Cell Lines

The *B. abortus* A19 vaccine strain was provided by the Shaanxi Veterinary Drug Supervision Institute (Shaanxi Province, China) and was grown on tryptic soy agar or tryptic soy broth at 37°C. The *sepA* deletion mutant (Δ*SepA*) and the *sepA* complemented strain (*C*‐Δ*SepA*) of *B. abortus* A19 were constructed and maintained in our laboratory. RAW264.7 macrophage cells and HEK293T cells (National Collection of Authenticated Cell Cultures, Shanghai, China) were cultured at 37°C in DMEM containing 10% fetal bovine serum (FBS) in a 5% CO_2_ incubator. RAW264.7 cells were used for *B. abortus* A19 infection assays, and HEK293T cells were used for dual‐luciferase assays. All experiments involving *B. abortus* A19 were performed exclusively in a Biosafety Level 2 (P2) laboratory. *B. abortus* A19 is legally BSL2 in China.

### 2.2. Construction of the *sepA* Deletion Mutant and Complemented Strain

To generate the *sepA* deletion mutant, the *sepA* gene was replaced with the kanamycin resistance gene (*KanR*) via homologous recombination, following established protocols [36]. Briefly, the upstream and downstream homologous sequences of the *sepA* gene from *Brucella abortus* A19, together with the *KanR* gene, were amplified and inserted into the pMD19T vector to construct the suicide plasmid pMD19T‐s*epA*. This plasmid was transformed into *B. abortus* A19 by electroporation, and kanamycin‐resistant colonies were screened to obtain the target strain, which was further verified by qPCR.

To generate the complemented strain of the *sepA* deletion mutant, we constructed the recombinant plasmid pBBR‐Amp‐sepA. This engineered vector retains the *pBBR1 oriV* and *pBBR1 Rep* regions from the parental plasmid pBBR1MCS‐5 and includes the 500‐bp upstream sequence of *sepA*, the full‐length *sepA* open reading frame, a *Flag* tag, and an *ampicillin* resistance cassette. The oligonucleotide primers used for assembly of pBBR‐Amp‐sepA, PBBori (*C*‐Δ*SepA*), SepA (*C*‐Δ*SepA*), and Amp (*C*‐Δ*SepA*) are detailed in Table 1, with specific designations and functions as follows: PBBori (*C*‐Δ*SepA*) was used to amplify the *pBBR1 oriV* and *pBBR1 Rep* backbone region; SepA (*C*‐Δ*SepA*) (reverse primer, incorporating the Flag tag in‐frame) and the forward primer were used to amplify the 500‐bp *sepA* upstream sequence fused to *sepA*‐*Flag*; and Amp (*C*‐Δ*SepA*) was used to amplify the *ampicillin* resistance cassette. The fragments were seamlessly assembled using the Seamless Assembly and Cloning Kit (DiNing, Beijing, China) to yield pBBR‐Amp‐sepA. This plasmid was transformed into the *sepA* deletion mutant, and ampicillin‐resistant colonies were screened. The complemented strain was verified by qPCR and Western blotting.

**Table 1:** Primers used in plasmid construction and qRT‐PCR.

Gene	Forward	Reverse
Slc2a1	5^′^‐AAGCTTATGTTGGCTGTGGGAGGAGCAGTGC‐3^′^	5^′^‐GGATCCTCACACTTGGGAGTCCGCCCCCAGA ‐ 3 ’
SepA	5^′^‐AAGCTTATGATGCCCGTGATTAGACT ‐ 3^′^	5^′^‐GGATCCTTAGGCGGACGCCGGGCCAG ‐ 3 ’
BspE	5^′^‐GCGGCCGCGATGACGTTATCGACGCGTAT‐3^′^	5^′^‐GGTACCTCAGGCAGCAACTTGCGATG‐3^′^
NyxA	5^′^‐AAGCTTATGAACGCTCACACAAACAT‐3^′^	5^′^‐GGATCCTCAAAGCTCCAAGCATCTAA‐3^′^
NyxB	5^′^‐AAGCTTATGAACACGCAAGCAACAAT ‐ 3^′^	5^′^‐GGATCCTCAAGGCATCTCGATAAGGC ‐ 3^′^
Slc2a1	5^′^‐AAGCTTATGTTGGCTGTGGGAGGAGCAGTGC‐3^′^	5^′^‐GGATCCTCACACTTGGGAGTCCGCCCCCAGA ‐ 3 ’
Fabp4	5^′^‐AAGGATGACGATGACAAGCTTATGTGTGATGCCTTTGTGGGAACCT‐3^′^	5^′^‐CAGATCTATCGATGAATTCTCATGCCCTTTCATAAACTCTTGTG‐ 3 ’
Pro −2000 to −1	5^′^‐GGCCTAACTGGCCGGTACCGAGAAACAGAGCACAGAGCACAGAG‐ 3 ’	5^′^‐AGGCTAGCGAGCTCAGGTACCGATAGCTCACTTTTAAAGATGCCCT‐ 3 ’
Pro −1500 to −1	5^′^‐GGCCTAACTGGCCGGTACCACATGTCTAAGTTAGGGATCAGCAT‐ 3 ’	5^′^‐AGGCTAGCGAGCTCAGGTACCGATAGCTCACTTTTAAAGATGCCCT‐ 3 ’
Pro −1000 to −1	5^′^‐GGCCTAACTGGCCGGTACCAGGTTTTGATAATTCATGACCACAT‐ 3 ’	5^′^‐AGGCTAGCGAGCTCAGGTACCGATAGCTCACTTTTAAAGATGCCCT‐ 3 ’
Pro −500 to −1	5^′^‐GGCCTAACTGGCCGGTACCACAACCAAAAATCAAAAAACTAGGC‐ 3 ’	5^′^‐AGGCTAGCGAGCTCAGGTACCGATAGCTCACTTTTAAAGATGCCCT‐ 3 ’
Pro −400 to −1	5^′^‐GGCCTAACTGGCCGGTACCAAGAATCTCTAAGATAGTTTTTATG‐ 3 ’	5^′^‐AGGCTAGCGAGCTCAGGTACCGATAGCTCACTTTTAAAGATGCCCT‐ 3 ’
Pro −300 to −1	5^′^‐GGCCTAACTGGCCGGTACCTGTCCAAGATAATTGTCTCCTCCAC‐ 3 ’	5^′^‐AGGCTAGCGAGCTCAGGTACCGATAGCTCACTTTTAAAGATGCCCT‐ 3 ’
Pro −200 to −1	5^′^‐GGCCTAACTGGCCGGTACCAAAATACTCTGTTCATGGTTAAAAA‐ 3 ’	5^′^‐AGGCTAGCGAGCTCAGGTACCGATAGCTCACTTTTAAAGATGCCCT‐ 3 ’
Pro −100 to −1	5^′^‐GGCCTAACTGGCCGGTACCAGAGGAAAACATACAGGGTCTGGTC‐ 3 ’	5^′^‐AGGCTAGCGAGCTCAGGTACCGATAGCTCACTTTTAAAGATGCCCT‐ 3 ’
Pro Nrih3 mut	5^′^‐CAGAGGAAAACATACAGGGTCTGGTCATGAAGGAAATGATCTGGCCCCCATTGGTCACTCCTACAGTCACATGGTCCTTATGTCTTTAAAAGTGAGCTATCA‐ 3 ’	5^′^‐AGCTTGATAGCTCACTTTTAAAGACATAAGGACCATGTGACTGTAGGAGTGACCAATGGGGGCCAGATCATTTCCTTCATGACCAGACCCTGTATGTTTTCCTCTGGTAC‐ 3 ’
Pro ELF5 mut	5^′^‐CAGAGGAAAACATACAGGGTCTGGTCATGTGTTTGTGCATCTGGCCCCCATTGGTCACTCCTACAGTCACATGGTCAGGGCATCTTTAAAAGTGAGCTATCA‐ 3 ’	5^′^‐AGCTTGATAGCTCACTTTTAAAGATGCCCTGACCATGTGACTGTAGGAGTGACCAATGGGGGCCAGATGCACAAACACATGACCAGACCCTGTATGTTTTCCTCTGGTAC‐ 3 ’
Pro Usf1/Usf2 mut	5^′^‐CAGAGGAAAACATACAGGGTCTGGTCATGAAGGAAATGATCTGGCCCCCATTGGTCACTCCTACAGTGCACGCTTCAGGGCATCTTTAAAAGTGAGCTATCA‐ 3 ’	5^′^‐AGCTTGATAGCTCACTTTTAAAGATGCCCTGAAGCGTGCACTGTAGGAGTGACCAATGGGGGCCAGATCATTTCCTTCATGACCAGACCCTGTATGTTTTCCTCTGGTAC‐ 3 ’
Usf1	5^′^‐AGCCCGGGCGGATCCAAGCTTATGAAGGGGCAGCAGAAAACAGCTG‐ 3 ’	AGATCTGTCGACGATATCGAATTCTTAGTTGCTGTCATTCTTGATGACG‐ 3 ’
Usf2	5^′^‐AGAGCCCGGGCGGATCCAAGCTTATGGACATGCTGGACCCGGGTCTGG‐ 3 ’	GATCTGTCGACGATATCGAATTCTCACTGCCGGGTACTCTCGCCCACC‐ 3 ’
qRT‐PCR‐Fabp4	5^′^‐CATAACCCTAGATGGCGGGG‐ 3 ’	5^′^‐CCAGCTTGTCACCATCTCGT‐ 3 ’
qRT‐PCR‐IL‐1β	5^′^‐TGCCACCTTTTGACAGTGATG‐ 3 ’	5^′^‐TGATGTGCTGCTGCGAGATT‐ 3 ’
qRT‐PCR‐IL‐6	5^′^‐CTGCAAGAGACTTCCATCCAG‐ 3 ’	5^′^‐AGTGGTATAGACAGGTCTGTTGG‐ 3 ’
qRT‐PCR‐iNOS	5^′^‐GTTCTCAGCCCAACAATACAAGA‐ 3 ’	5^′^‐GTGGACGGGTCGATGTCAC‐ 3 ’
qRT‐PCR‐TNF‐α	5^′^‐GGGGATACATCCATCAGGGGT‐ 3 ’	5^′^‐GCTCGGACAGTCACTCACC‐ 3 ’
qRT‐PCR‐TGF‐β	5^′^‐CCACCTGCAAGACCATCGAC‐ 3 ’	5^′^‐CTGGCGAGCCTTAGTTTGGAC‐ 3 ’
qRT‐PCR‐ARG1	5^′^‐CTCCAAGCCAAAGTCCTTAGAG‐ 3 ’	5^′^‐GGAGCTGTCATTAGGGACATCA‐ 3 ’
qRT‐PCR‐β‐actin	5^′^‐GGCTGTATTCCCCTCCATCG‐ 3 ’	5^′^‐CCAGTTGGTAACAATGCCATGT‐ 3 ’
UP1000 (Δ*SepA*)	5^′^‐CAGGTCGACTCTAGAGGATCCCCCTCGGTTCAAATTGAAA‐ 3 ’	5^′^‐AGAACTCTGTAGCACCGCGGCTCTCTCCGCTCAAGG‐ 3 ’
KanR (Δ*SepA*)	5^′^‐GCCGCGGTGCTACAGAGTTCT‐ 3 ’	5^′^‐CCTTTAACAAAAATTTAACGC‐ 3 ’
DOWN900 (Δ*SepA*)	5^′^‐GCGTTAAATTTTTGTTAAAGGTCACGCAGGCAGCTT‐ 3 ’	5^′^‐ACGACGGCCAGTGAATTCTCCAAGCCCCTCCAGCGC‐ 3 ’
PBBori (*C*‐Δ*SepA*)	5^′^‐TGGTAATTCGCAAAGTCGTGACCGCCTACGGCG‐ 3 ’	5^′^‐ACTGGAAGACCTCCCAGAGCCTGATAAAAACGG‐ 3 ’
SepA (*C*‐Δ*SepA*)	5^′^‐ATCAGGCTCTGGGAGGTCTTCCAGTCAACGACTTGGGCGAA‐ 3 ’	5^′^‐TTACTTGTCATCGTCGTCCTTGTAATCGGCGGACGCCGGGCCAGAC‐ 3 ’
Amp (*C*‐Δ*SepA*)	5^′^‐GATTACAAGGACGACGATGACAAGTAACGCGGAACCCCTATTTGTTTA‐ 3 ’	5^′^‐CGGTCACGACTTTGCGAATTACCAATGCTTAATCAGTGAGGC‐ 3 ’
qRT‐PCR‐sepA	5^′^‐CCCGAGAGGCCTTTTGTCTT‐ 3 ’	5^′^‐TCGACGGCAAGGCATTACAT‐ 3 ’
Slc2a1 Pro −2000 to −1	GAACATTTCTCTGGCCTAACTGGCCGGTACCGGGACTGGGCTGAAATGCTGA	GATATCCTCGAGGCTAGCGAGCTCAGGTACCGGGGGAGAGCGCGCGGAGC
Slc2a1 Pro −1500 to −1	GAACATTTCTCTGGCCTAACTGGCCGGTACCGAGCCACACATACACACACAC	GATATCCTCGAGGCTAGCGAGCTCAGGTACCGGGGGAGAGCGCGCGGAGC
Slc2a1 Pro −1000 to −1	GAACATTTCTCTGGCCTAACTGGCCGGTACCGGTCCATGCTCAATTAGGGTG	GATATCCTCGAGGCTAGCGAGCTCAGGTACCGGGGGAGAGCGCGCGGAGC
Slc2a1 Pro −500 to −1	GAACATTTCTCTGGCCTAACTGGCCGGTACCGAGCCAGACCTCCCGTGGTCT	GATATCCTCGAGGCTAGCGAGCTCAGGTACCGGGGGAGAGCGCGCGGAGC
Slc2a1 Pro −500 to −300	GAACATTTCTCTGGCCTAACTGGCCGGTACCGAGCCAGACCTCCCGTGGTCT	TCCTCGAGGCTAGCGAGCTCAGGTACCCGGGGCTGTAAGCCAGCGCTA
Slc2a1 Pro −300 to −1	ATTTCTCTGGCCTAACTGGCCGGTACCCACCAAGCGCTACCATTTTGC	GATATCCTCGAGGCTAGCGAGCTCAGGTACCGGGGGAGAGCGCGCGGAGC
Slc2a1 Pro −150 to −1	ATTTCTCTGGCCTAACTGGCCGGTACCCCTCCGTCCCTCCCCACCTCC	GATATCCTCGAGGCTAGCGAGCTCAGGTACCGGGGGAGAGCGCGCGGAGC

### 2.3. Cell Infection Assay

RAW264.7 cells were gently detached from culture dishes by pipetting with fresh culture medium, counted using a cell counting chamber, and then seeded at 2 × 10^5^ per well in 24‐well plates and cultured at 37°C in 5% CO_2_ for 12 h. *B. abortus* A19 was added at a multiplicity of infection (MOI) of 200 and incubated for 4 h. After removing the inoculum, the monolayers were rinsed three times with sterile PBS, and cells were then incubated with cell culture medium containing gentamicin (50 µg/mL) for 1 h to eliminate extracellular bacteria. After discarding the medium and washing three times with PBS, fresh medium supplemented with gentamicin (25 µg/mL) was added (time point = 0 h postinfection [hpi]).

### 2.4. Plasmids

The eukaryotic expression plasmids used in this study included pCMV‐3×Flag‐Slc2a1, pCMV‐3×Flag‐SepA, pCMV‐3×Flag‐BspE, pCMV‐3×Flag‐NyxA, pCMV‐3×Flag‐NyxB, pCMV‐Fabp4‐3×Flag, pCMV‐USF1‐HA, pCMV‐USF2‐3×Flag, and the corresponding empty vector controls. Dual‐luciferase reporter plasmids included pGL4.10, pGL4.10‐promoter(−2000 to −1), pGL4.10‐promoter(−1500 to −1), pGL4.10‐promoter(−1000 to −1), pGL4.10‐promoter(−500 to −1), pGL4.10‐promoter(−400 to −1), pGL4.10‐promoter(−300 to −1), pGL4.10‐promoter(−200 to −1), pGL4.10‐promoter(−100 to −1), pGL4.10‐Nrih3mut, pGL4.10‐ELF5mut, pGL4.10‐Usf1/Usf2mut, and pRL‐4.10. All inserted sequences in the dual‐luciferase reporter plasmids were derived from mouse genomic DNA. The specific primer sequences for plasmid construction are detailed in Table 1.

### 2.5. Detection of Intracellular L‐Lactate Content

Intracellular L‐lactate levels were measured using the lactic acid (LA) content Assay Kit (Solarbio, Beijing, China) according to the manufacturer’s instructions. Briefly, cells were resuspended in Extraction Buffer I at a density of 5 × 10^6^ cells/mL and lysed by ice‐bath ultrasonication (300 W, 3 s pulse on/7 s pulse off, total duration: 3 min). The lysate was centrifuged at 12,000 × *g* for 10 min at room temperature. The supernatant (0.8 mL) was collected, and 0.15 mL of Extraction Buffer II was added slowly with gentle pipetting to ensure thorough mixing without introducing bubbles. The mixture was centrifuged again at 12,000 × *g* for 10 min at room temperature. The resulting supernatant was used for the spectrophotometric determination of L‐lactate concentration at 570 nm.

### 2.6. Liquid Chromatography‐Mass Spectrometry Data Analysis

Functional annotation of proteins was performed using InterProScan, which mapped Gene Ontology (GO) terms and InterPro (IPR) domains against nonredundant protein databases, including Pfam, PRINTS, ProDom, SMART, ProSite, and PANTHER. Additionally, protein family classification and pathway enrichment analyses were conducted using the Clusters of Orthologous Groups (COG) database and the Kyoto Encyclopedia of Genes and Genomes (KEGG) database, respectively. The differentially expressed proteins were utilized to generate volcano plots, cluster heat maps, and enrichment analyses for GO, IPR, and KEGG categories.

### 2.7. Quantitative Real‐Time PCR

Total RNA was extracted from RAW264.7 cells and reverse‐transcribed into cDNA using the PrimeScript RT Reagent Kit (TaKaRa, Tokyo, Japan). qRT‐PCR was then performed using the ChamQ SYBR qPCR Master Mix Kit (Vazyme, Nanjing, China) on a Bio‐Rad CFX96 Real‐Time PCR System. The β‐actin gene was used as the internal reference, and relative mRNA expression levels of target genes were quantified using the 2^−ΔΔCT^ computational method. The specific primer sequences used for qRT‐PCR are detailed in Table 2.

**Table 2:** siRNA and probe used in this work.

Gene	Forward	Reverse
siNC	5^′^‐UUCUCCGAACGUGUCACGUTT‐3^′^	5^′^‐ACGUGACACGUUCGGAGAATT‐3^′^
siFabp4‐111	5^′^‐GUCGACCACAAUAAAGAGATT‐3^′^	5^′^‐UCUCUUUAUUGUGGUCGACTT‐3^′^
siFabp4‐173	5^′^‐CCAAGCCCAACAUGAUCAUTT‐3^′^	5^′^‐AUGAUCAUGUUGGGCUUGGTT‐3^′^
siFabp4‐299	5^′^‐GGAAGGUGAAGAGCAUCAUTT‐3^′^	5^′^‐AUGAUGCUCUUCACCUUCCTT‐3^′^
siFabp4‐432	5^′^‐GACUUCCACAAGAGUUUAUTT‐3^′^	5^′^‐AUAAACUCUUGUGGAAGUCTT‐3^′^
siSlc2a1‐526	5^′^‐GCUUCAUCAUCGGUGUGUATT‐3^′^	5^′^‐UACACACCGAUGAUGAAGCTT‐3^′^
siSlc2a1‐820	5^′^‐CCAAGAGUGUGCUGAAGAATT‐3^′^	5^′^‐UUCUUCAGCACACUCUUGGTT‐3^′^
siSlc2a1‐1121	5^′^‐GCUGUUUGUUGUAGAGCGATT‐3^′^	5^′^‐UCGCUCUACAACAAACAGCTT‐3^′^
siSlc2a1‐1470	5^′^‐CUCGUGCUCUUCUUCAUCUTT‐3^′^	5^′^‐AGAUGAAGAAGAGCACGAGTT‐3^′^
Probe‐USF1/USF2	5^′^‐CACTCCTACAGTCACATGGTCAGGGCATCTTT‐3^′^	5^′^‐AAAGATGCCCTGACCATGTGACTGTAGGAGTG‐3^′^
Probe‐USF1/USF2‐CY3	5^′^‐CACTCCTACAGTCACATGGTCAGGGCATCTTT‐3^′^	5^′^‐CY3‐AAAGATGCCCTGACCATGTGACTGTAGGAGTG‐3^′^
Probe‐USF1/USF2‐Mut	5^′^‐CACTCCTACAGTGCACGCTTCAGGGCATCTTI‐3^′^	5^′^‐AAAGATGCCCTGAAGCGTGCACTGTAGGAGTG‐3^′^

### 2.8. Western Blotting

RAW264.7 cells were lysed using RIPA lysis buffer (Beyotime, Shanghai, China), and the total protein concentration was determined using the BCA Protein Assay Kit (Solarbio, Beijing, China). Ten micrograms of protein per sample were separated by 12.5% SDS‐PAGE. Electrophoresis was initially performed at a constant voltage of 80 V for 30 min, followed by 120 V for 60 min. Proteins were then transferred to a PVDF membrane. The membrane was blocked with TBST containing 10% skim milk for 2 h at room temperature and subsequently incubated for 2 h at room temperature with primary antibodies diluted in TBST containing 10% skim milk (or antibody dilution buffer): anti‐L‐lactyl lysine (PTM BIO, Hangzhou, China), anti‐Flag (CST, Boston, MA, USA), anti‐Slc2a1 (Proteintech, Wuhan, China), anti‐FABP4 (Proteintech), anti‐β‐actin (Proteintech), or anti‐GAPDH (Proteintech). After washing with TBST to remove unbound primary antibodies, the membrane was incubated with HRP‐conjugated rabbit secondary antibody (Beijing CWBIO, Beijing, China) for 1–2 h at room temperature. Following final washes with TBST, protein bands were visualized using enhanced chemiluminescence (ECL) detection.

### 2.9. RNA Interference

siRNAs targeting Slc2a1 and Fabp4 were designed and synthesized by GenePharma (Shanghai, China) (Table 1). The appropriate siRNA was transfected into RAW264.7 cells using the Advanced DNA RNA Transfection Reagent (Zeta Life, New York, NY, USA) according to the manufacturer’s instructions. Twenty‐four hours after transfection, cells were infected with *B. abortus* A19 at a MOI of 200. Subsequent experiments involving *B. abortus* A19 infection were performed as described above.

### 2.10. Coimmunoprecipitation Analysis

RAW264.7 cells were seeded in 60‐mm cell culture plates and grown to ~70% cell density before transfection. Cells were transfected with pCMV‐USF2‐3×Flag or the empty vector pCMV‐MCS‐3×Flag using Lipofectamine 2000 (Invitrogen, Carlsbad, CA, USA) according to the manufacturer’s instructions. Twenty‐four hours post‐transfection, cells were treated with 10 mM L‐lactate for an additional 24 h. Cell lysates were prepared, and immunoprecipitation was performed using the BeaverBeads Protein A (or Protein A/G) Immunoprecipitation Kit (BEAVER, Suzhou, China) following the manufacturer’s protocol. Briefly, antibodies were coupled to magnetic beads, and the beads were incubated with cell lysates overnight at 4°C with gentle rotation. After washing, bound proteins were eluted, and the lactylation levels of USF2 were detected by Western blotting.

### 2.11. Dual‐Luciferase Reporter Assay

Dual‐luciferase reporter assays were performed using the Dual‐Luciferase Reporter Assay System (Promega, Madison, WI, USA) according to the manufacturer’s instructions. For Fabp4 promoter reporter assays, HEK293T cells seeded in 24‐well plates were cotransfected with 200 ng of the Fabp4 promoter luciferase reporter plasmid and 50 ng of pRL‐CMV (Renilla luciferase control) using Lipofectamine 2000 (Invitrogen, Carlsbad, CA, USA). Twenty‐four hours post‐transfection, cells were lysed, and firefly and Renilla luciferase activities were measured sequentially using a Tecan Spark multimode microplate reader. Firefly luciferase activity was normalized to Renilla luciferase activity to correct for transfection efficiency.

### 2.12. Electrophoretic Mobility Shift Assay (EMSA)

RAW264.7 cells were seeded in 60‐mm cell culture plates and grown to ~70% density before transfection with pCMV‐USF1‐HA or pCMV‐USF2‐3×Flag using Lipofectamine 2000 (Invitrogen, Carlsbad, CA, USA). Forty‐eight hours post‐transfection, cells were harvested, and nuclear proteins were extracted using the Nuclear and Cytoplasmic Protein Extraction Kit (Beyotime, Shanghai, China) according to the manufacturer’s instructions. Cy3‐labeled fluorescent probes (Table 2) were synthesized by Tsingke Biotechnology (Beijing, China). EMSAs were performed using the EMSA/Gel Shift Kit (Beyotime, Shanghai, China) following the manufacturer’s protocol.

### 2.13. Lipid Droplet Detection

Lipid droplets in RAW264.7 cells were stained using the Nile Red Staining Kit (Beyotime, Shanghai, China) according to the manufacturer’s instructions. Briefly, cells were seeded on cell slides, infected with *B. abortus* A19 at an MOI of 200 for 24 h, fixed with 4% paraformaldehyde at room temperature for 10–15 min, and washed with PBS. Nile Red dye was added, and cells were incubated in the dark at room temperature for 10–20 min. Fluorescence was observed under a fluorescence microscope with excitation at 552 nm for red fluorescence and emission in the red channel (>590 nm).

### 2.14. Statistical Analysis

All data are presented as mean ± standard deviation (SD) from at least three independent experiments. For comparisons between two groups, a two‐tailed unpaired Student’s *t*‐test was used. For comparisons among three or more groups, one‐way ANOVA was performed, followed by Tukey’s HSD test for all pairwise comparisons or Dunnett’s test for comparisons of each experimental group with the control group, according to the predefined comparison of interest. Statistical significance was defined as  ^∗^ *p*  < 0.05 (statistically significant),  ^∗∗^ *p*  < 0.01 (highly statistically significant),  ^∗∗∗^ *p*  < 0.001 (very highly statistically significant), and  ^∗∗∗∗^ *p*  < 0.0001 (extremely statistically significant differences).

## 3. Results

### 3.1. L‐Lactate Enhances Intracellular Proliferation of *B. abortus* in Macrophages

RAW264.7 cells were infected with *B. abortus* A19. Quantitative analysis demonstrated significant upregulation of intracellular L‐lactate levels in infected cells at 24 hpi compared with uninfected controls (Figure 1A). In addition, a time‐dependent elevation in cellular lactylation levels was observed in infected cells, with significantly higher lactylation observed in infected cells compared with uninfected controls at both 12 and 24 hpi (Figure 1B).

**Figure 1:**
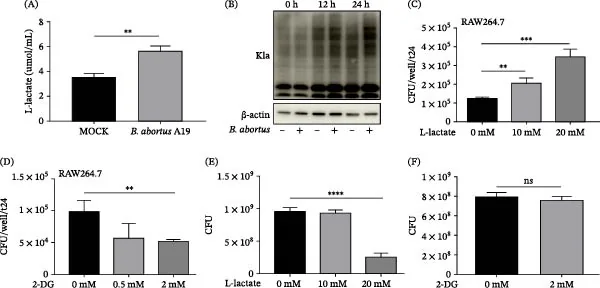
L‐lactate enhances intracellular proliferation of *B. abortus* in RAW264.7 macrophages. (A) At 24 h postinfection, intracellular L‐lactate levels were measured in RAW264.7 cells infected with *B. abortus* (MOI of 200) and compared with those in uninfected control cells. (B) Global lactylation levels in RAW264.7 cells infected with *B. abortus* were analyzed by Western blotting at the indicated time points (0, 12, and 24 hpi). (C) RAW264.7 cells infected with *B. abortus* were treated with 0, 10, or 20 mM L‐lactate, and intracellular bacterial burden was quantified by CFU assay at 24 h posttreatment. The 10 and 20 mM L‐lactate groups were compared with the 0 mM control group. (D) Infected RAW264.7 cells were treated with 0, 0.5, or 2 mM 2‐DG, and bacterial load was assessed by CFU assay at 24 h posttreatment. Bacterial loads in the 0.5 and 2 mM 2‐DG groups were compared with those in the 0 mM control group. (E) *B. abortus* was cultured in TSB supplemented with 0, 10, or 20 mM L‐lactate, and bacterial viability (CFU) was measured after 24 h. (F) *B. abortus* was grown in TSB containing 0 or 2 mM 2‐DG, and bacterial proliferation (CFU) was evaluated at 24 h postinoculation. Experiments were repeated at least three times with similar results. Data are presented as mean ± SD from three independent experiments (*n* = 3 per group). For comparisons between two groups, a two‐tailed unpaired Student’s *t*‐test was used. For comparisons among three or more groups, one‐way analysis of variance (ANOVA) followed by Dunnett’s post hoc test was performed to compare each experimental group with the control group.  ^∗∗^ *p*  < 0.01;  ^∗∗∗^ *p*  < 0.001;  ^∗∗∗∗^ *p*  < 0.01.

To determine whether exogenous L‐lactate influences bacterial proliferation, *B. abortus*‐infected RAW264.7 cells were treated with varying concentrations of L‐lactate. A significant enhancement of intracellular bacterial burden was observed at 24 hpi following treatment with 10 or 20 mM L‐lactate, whereas no significant difference was detected at 2 hpi (Figures 1C and S1A). L‐lactate is the end product of glycolysis, and its production can be attenuated by glycolytic inhibitors. We therefore treated infected RAW264.7 cells with the glycolytic inhibitor 2‐deoxy‐D‐glucose (2‐DG). Treatment with 2 mM 2‐DG had no significant effect on bacterial survival at 2 hpi but markedly inhibited its intracellular proliferation at 24 hpi (Figures 1D and S1B). To exclude the possibility that the effects of L‐lactate or 2‐DG on intracellular bacterial proliferation were due to direct bactericidal activity, we examined bacterial growth in TSB supplemented with these compounds. L‐lactate (10 or 20 mM) inhibited *B. abortus* proliferation in vitro, whereas 2‐DG (2 mM) showed no inhibitory effect under the same conditions (Figure 1E,F). Collectively, these results indicate that *B. abortus* infection induces elevated L‐lactate production in macrophages, which in turn promotes intracellular bacterial proliferation.

### 3.2. *B. abortus* Modulates Host Glycolytic Protein Slc2a1 via Effector SepA to Promote Cellular Lactylation in Macrophages

To investigate the mechanism by which *B. abortus* stimulates L‐lactate production and protein lactylation in macrophages, we performed comparative proteomic analysis of infected and uninfected RAW264.7 cells. Upregulation of Slc2a1, Pgm2, Eno2, Hk1, and Pfkp was observed following *B. abortus* infection compared to their expression in uninfected cells in the heat map. Among these, Slc2a1 exhibited the most pronounced differential expression (Figure 2A). This observation was assessed further by Western blotting, which demonstrated a consistent elevation of Slc2a1 in infected cells (Figure 2B,C). An *SLC2A1* overexpression vector and siRNA that targeted the gene were constructed to further investigate the role of the protein in the intracellular proliferation of *B. abortus* in macrophages. Overexpression of *SLC2A1* facilitated *B. abortus* replication, whereas knockdown of *SLC2A1* markedly inhibited its intracellular proliferation at 24 hpi (Figure 2D–F). Neither overexpression nor knockdown of *SLC2A1* affected the intracellular survival of *B. abortus* at 2 hpi (Figure S2A,B). We also found that Slc2a1 knockdown significantly suppressed the capacity of *B. abortus* to induce L‐lactate production in RAW264.7 cells (Figure S1C). Moreover, overexpression of *SLC2A1* in RAW264.7 cells significantly enhanced lactylation levels, whereas knockdown of the gene markedly suppressed protein lactylation (Figure 2G,H).

**Figure 2:**
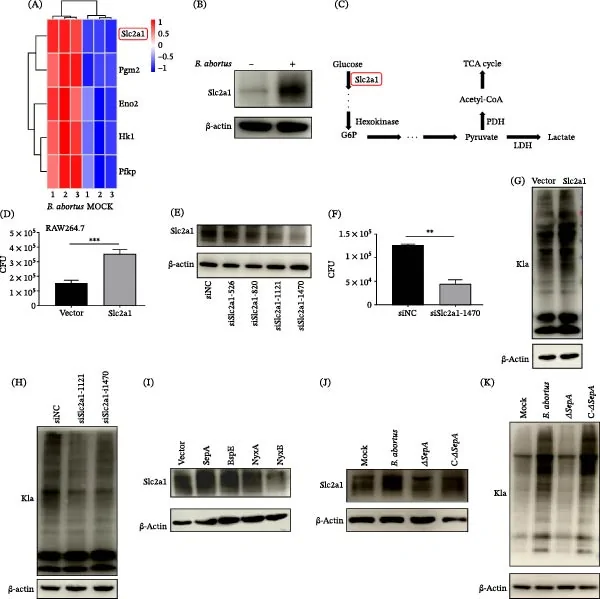
*B. abortus* modulates host glycolytic protein Slc2a1 via effector SepA to promote cellular lactylation in RAW264.7 macrophages. (A) Heat map showing protein levels of Slc2a1, Pgm2, Eno2, Hk1, and Pfkp in uninfected RAW264.7 cells and cells infected with *B. abortus* for 24 h. Differentially abundant proteins were selected using |log_2_FC| ≥ 1 and FDR < 0.05. The color scale represents row‐scaled *z*‐scores of log_2_‐transformed protein intensities, with red indicating higher abundance and blue indicating lower abundance. (B) Western blotting analysis of Slc2a1 protein levels in uninfected and *B. abortus*‐infected RAW264.7 cells at 24 hpi. (C) Schematic overview of glucose metabolism highlighting key enzymes. (D) RAW264.7 cells were transfected with p3×FLAG‐CMV or p3 × FLAG‐CMV‐Slc2a1 for 24 h, then infected with *B. abortus* (MOI = 200). Intracellular bacterial CFU were determined at 24 hpi and compared between the p3×FLAG‐CMV‐Slc2a1 and p3×FLAG‐CMV groups. (E) RAW264.7 cells were transfected with negative control siRNA (siNC) or Slc2a1‐specific siRNAs (siSlc2a1‐520, ‐820, ‐1121, or ‐1470; 50 nM) for 24 h, and Slc2a1 protein levels were assessed by Western blotting. (F) RAW264.7 cells transfected with siNC or siSlc2a1‐1470 (50 nM) for 24 h were infected with *B. abortus* (MOI = 200). Intracellular bacterial CFU were quantified at 24 hpi and compared between the siSlc2a1‐1470 and siNC groups. (G, H) Following Slc2a1 overexpression or knockdown, RAW264.7 cells were infected with *B. abortus* for 24 h, and global lactylation levels were analyzed by Western blotting. (I) RAW264.7 cells were transfected with p3×FLAG‐CMV, p3×FLAG‐CMV‐SepA, p3×FLAG‐CMV‐BspE, p3×FLAG‐CMV‐NyxA, or p3×FLAG‐CMV‐NyxB for 24 h, followed by detection of Slc2a1 expression. (J, K) RAW264.7 cells were infected with *B. abortus*, Δ*SepA* mutant, or complemented strain (*C*‐Δ*SepA*) for 24 h, and Slc2a1 protein levels and lactylation status were analyzed. Data are presented as mean ± SD from three independent experiments (*n* = 3 per group). For comparisons between two groups, a two‐tailed unpaired Student’s *t*‐test was used. Experiments were repeated at least three times with similar results.  ^∗∗^ *p*  < 0.01;  ^∗∗∗^ *p*  < 0.001.

Given the critical role of bacterial effector proteins in intracellular survival, we hypothesized that one or more of these proteins in *B. abortus* may impact the expression of *SLC2A1* in macrophages. Therefore, we constructed eukaryotic expression plasmids that encoded several key *Brucella* effector proteins and transfected these constructs into RAW264.7 cells. Effector protein SepA upregulated *SLC2A1* expression, whereas the expression of the genes for effectors BspE, NyxA, and NyxB did not impact *SLC2A1* (Figure 2I). The role of SepA in modulating *SLC2A1* expression was investigated further with a *B. abortus sepA* gene deletion mutant (Δ*SepA*) and *sepA*‐complemented strain (*C*‐Δ*SepA*). This mutant was significantly impaired in the induction of *SLC2A1* expression (Figure 2J). Furthermore, we observed that deletion of the gene significantly reduced the ability of *B. abortus* to induce host cell lactylation (Figure 2K).

To investigate the mechanism by which SepA regulates Slc2a1 expression, we generated a Slc2a1 promoter‐driven dual‐luciferase reporter plasmid and found that SepA upregulated the Slc2a1 promoter activity (Figure S6A,B). To further delineate the core promoter region of Slc2a1, we performed promoter truncation analysis and mapped the core promoter region to the interval from −150 to −1 (Figure S6C,D). Using AnimalTFDB v4.0, Vezf1 was predicted to be a putative transcription factor (Figure S6E). Subsequently, by constructing dual‐luciferase reporter plasmids harboring mutations in the Vezf1 binding site and a *Vezf1* overexpression plasmid, we demonstrated that Vezf1 regulates the Slc2a1 expression (Figure S6F–H). Relative to the Δ*SepA* mutant, infection of RAW264.7 cells with either *B. abortus* or the *sepA*‐complemented strain elicited an elevated Vezf1 expression (Figure S6I).

### 3.3. L‐Lactate Enhances Proliferation of *B. abortus* in Macrophages via Suppression of Host FABP4 Protein

The preceding data demonstrated that *B. abortus* upregulates *SLC2A1* expression to facilitate L‐lactate synthesis. The mechanism by which L‐lactate modulates intracellular proliferation was explored by comparative proteomic profiling of macrophages that were infected with *B. abortus* and treated with L‐lactate simultaneously. L‐lactate exerted significant regulatory effects on cellular metabolic activity by modulating the levels of multiple metabolism‐associated proteins. Comprehensive proteomic analysis and Western blotting revealed that FABP4, a key lipid metabolism‐associated protein, was upregulated upon *B. abortus* infection, but this pathogen‐induced upregulation was suppressed by L‐lactate treatment (Figure 3A–C). To determine whether *FABP4* influences bacterial intracellular proliferation, we constructed an overexpression plasmid and tested its effect. Overexpression of FABP4 significantly inhibited the intracellular proliferation of *B. abortus*, the Δ*SepA* mutant, and the complemented strain (*C*‐Δ*SepA*) at 24 hpi (Figures 3D and S3A,B), whereas bacterial survival at 2 hpi remained unaffected (Figure 3E). Subsequently, we designed and synthesized siRNAs that targeted *FABP4*, among which siFabp4‐432 exhibited the highest knockdown efficiency (Figure 3F,G). Further experiments utilizing this siRNA revealed that *FABP4* knockdown markedly promoted the intracellular proliferation of *B. abortus*, Δ*SepA*, and *C*‐Δ*SepA* at 24 hpi (Figures 3H and S3C,D); the intracellular survival of *B. abortus* remained unaffected at 2 hpi (Figure 3I). In summary, these results demonstrate that L‐lactate enhances the proliferation of *B. abortus* in macrophages via the suppression of host FABP4 protein.

**Figure 3:**
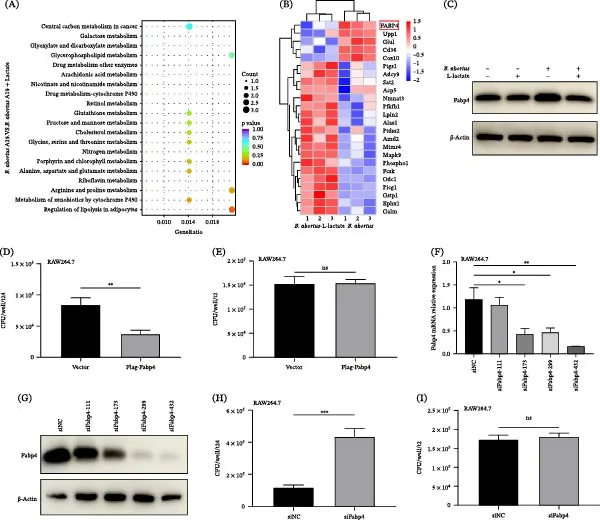
L‐lactate enhances intracellular proliferation of *B. abortus* via suppression of FABP4 in RAW264.7 macrophages. (A, B) KEGG enrichment analysis and heat map of metabolism‐related signaling pathways and differentially expressed proteins in *B. abortus*‐infected RAW264.7 cells with or without L‐lactate treatment. Differentially abundant proteins were selected using |log_2_FC| ≥ 1 and FDR < 0.05. The color scale represents row‐scaled *z*‐scores of log_2_‐transformed protein intensities, with red indicating higher abundance and blue indicating lower abundance. (C) Western blotting analysis of Fabp4 protein levels in RAW264.7 cells following *B. abortus* infection and/or L‐lactate treatment. (D, E) RAW264.7 cells transfected with p3×FLAG‐CMV or p3×FLAG‐CMV‐Fabp4 were infected with *B. abortus* (MOI = 200). Intracellular bacterial loads were quantified at 24 hpi (D) and 2 hpi (E) and compared between the p3×FLAG‐CMV‐Fabp4 and p3×FLAG‐CMV groups at each time point. (F, G) RAW264.7 cells were transfected with negative control siRNA (siNC) or Fabp4‐specific siRNAs (siFabp4‐111, ‐173, ‐229, or ‐432; 50 nM) for 24 h, followed by qRT‐PCR and Western blotting to evaluate knockdown efficiency. (H, I) RAW264.7 cells transfected with siNC or siFabp4‐432 (50 nM) were infected with *B. abortus* (MOI = 200). Intracellular bacterial loads were quantified at 24 hpi (H) and 2 hpi (I) and compared between the siFabp4‐432 and siNC groups at each time point. Data are presented as mean ± SD from three independent experiments (*n* = 3 per group). For comparisons between two groups, a two‐tailed unpaired Student’s *t*‐test was used. For comparisons among three or more groups, one‐way analysis of variance (ANOVA) followed by Dunnett’s post hoc test was performed to compare each experimental group with the control group. Experiments were repeated at least three times with similar results.  ^∗^ *p*  < 0.05;  ^∗∗^ *p*  < 0.01;  ^∗∗∗^ *p*  < 0.001.

### 3.4. Host Transcription Factor USF2 Modulates *FABP4* Promoter Activity

Lactylation modification alters the biological activities of both histones and numerous nonhistone proteins [28–33]. We hypothesized that L‐lactate may regulate FABP4 expression via lactylation‐mediated modification of transcription factors that target the cognate promoter. Therefore, we constructed dual‐luciferase reporter plasmids containing full‐length and truncated *FABP4* promoter fragments of varying lengths. First, the functional regulatory elements of the promoter were mapped to the genomic interval that spans from −500 to −1 bp upstream of the transcription start site (Figure 4A). L‐lactate exerted a significant inhibitory effect on the activity of the dual‐luciferase reporter plasmid bearing this region (Figure 4B). Second, progressive truncation analysis identified that the core *FABP4* promoter region was localized in the −100 to −1 segment upstream of the transcription start site. L‐lactate also demonstrated significant suppression of the dual‐luciferase reporter activity in this critical region (Figure 4C,D).

**Figure 4:**
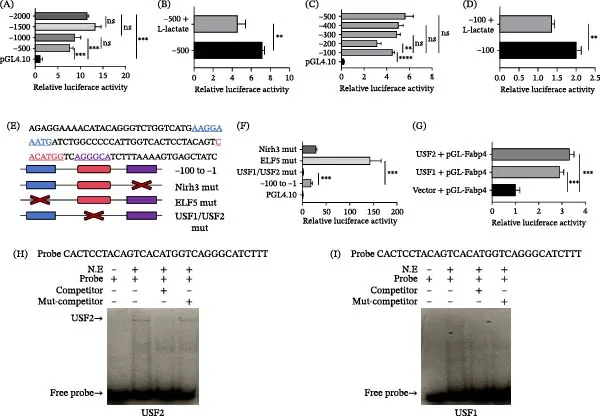
The transcription factor USF2 modulates the promoter activity of *FABP4*. (A) HEK293T cells were cotransfected with pRL‐CMV (Renilla control) and pGL4.10‐based reporter plasmids containing the indicated *FABP4* promoter fragments (pGL‐pro‐2000 to ‐1, ‐1500 to ‐1, ‐1000 to ‐1, or ‐500 to ‐1). Dual‐luciferase assays were performed 24 h post‐transfection to assess promoter activity. (B) HEK293T cells cotransfected with pRL‐CMV and pGL‐pro‐500 to −1 were treated with L‐lactate (10 mM) for 24 h, followed by dual‐luciferase reporter assays to measure *FABP4* promoter activity. (C) Progressive truncation analysis: HEK293T cells were cotransfected with pRL‐CMV and deletion constructs (pGL‐pro‐500 to −1, −400 to −1, −300 to −1, −200 to −1, or −100 to −1), and promoter activity was quantified by dual‐luciferase assays after 24 h. (D) HEK293T cells cotransfected with pRL‐CMV and pGL‐pro‐100 to −1 were treated with L‐lactate or untreated for 24 h. *FABP4* promoter activity was assessed and compared between the L‐lactate‐treated and untreated groups. (E) Computational prediction of transcription factor binding sites in the −100 to −1 region of the *FABP4* promoter using AnimalTFDB v4.0. (F) Site‐directed mutagenesis: HEK293T cells were cotransfected with pRL‐CMV and wild‐type or mutant pGL‐pro‐100 to −1 constructs (Nr1h3 mut, ELF5 mut, USF1/USF2 mut), and promoter activity was compared via dual‐luciferase assays. (G) HEK293T cells were cotransfected with pRL‐CMV, pGL‐pro‐100 to −1, and either pCMV‐USF1‐HA or pCMV‐USF2‐3×Flag. *FABP4* promoter activation was measured 24 h after transfection and compared among the pCMV‐USF1‐HA, pCMV‐USF2‐3×Flag, and pCMV empty vector control groups. (H, I) EMSA using nuclear extracts from RAW264.7 cells overexpressing HA‐USF1 or 3×Flag‐USF2 and Cy3‐labeled probes corresponding to the core *FABP4* promoter region (with or without competitors/antibodies for specificity). Experiments were repeated at least three times with similar results. Data are presented as mean ± SD from three independent experiments (*n* = 3 per group). For comparisons between two groups, a two‐tailed unpaired Student’s *t*‐test was used. For comparisons among three or more groups, one‐way analysis of variance (ANOVA) followed by Tukey’s HSD post hoc test for all pairwise comparisons was performed.  ^∗^ *p*  < 0.05;  ^∗∗^ *p*  < 0.01;  ^∗∗∗^ *p*  < 0.001;  ^∗∗∗∗^ *p*  < 0.0001.

Computational prediction using AnimalTFDB v4.0 revealed that USF1, USF2, Nrih3, and ELF5 were putative transcriptional regulators of *FABP4*. Site‐directed mutagenesis of the predicted binding motifs for these candidate factors in the −100 to −1 region of the *FABP4* promoter showed complete abolition of *FABP4* promoter‐driven luciferase activity following disruption of the USF1 and USF2 binding sites but not of the Nrih3 and ELF5 motifs (Figure 4E,F). Furthermore, ectopic overexpression of *USF1* and *USF2* from plasmid constructs resulted in significant potentiation of the *FABP4* promoter activity in the dual‐luciferase reporter system (Figure 4G). Subsequent EMSAs to investigate whether USF1 or USF2 interacted directly with the core *FABP4* promoter region demonstrated that USF2 exhibited specific binding activity, whereas USF1 failed to interact detectably with the promoter under identical experimental conditions (Figure 4H,I). Thus, the data strongly suggest that USF2 promotes transcription from the *FABP4* promoter.

### 3.5. *B. abortus* Modulates Lysine Lactylation of Transcription Factor USF2

The preceding results demonstrated that L‐lactate inhibited the promoter activity and expression of *FABP4* and that USF2 interacted directly with the core *FABP4* promoter region. We hypothesized that L‐lactate may regulate *FABP4* expression via the lactylation modification of the USF2 transcription factor. Global lactylation levels in RAW264.7 macrophages increased progressively during prolonged infection with *B. abortus*. Exogenous L‐lactate treatment further enhanced cellular lactylation levels (Figure 5A). Moreover, infection with *B. abortus* or *C*‐Δ*sepA* enhanced the lysine lactylation modification of USF2, whereas the impact of Δ*sepA* infection on USF2 lysine lactylation modification was substantially less pronounced than that of the former two. Although detectable USF2 lysine lactylation was also observed in uninfected macrophages, modification was substantially stronger in *B. abortus*‐infected cells (Figure 5B). Furthermore, comparative analysis showed that L‐lactate‐treated cells exhibited higher USF2 lysine lactylation levels relative to their untreated counterparts (Figure 5C). In general, lysine residues in proteins serve as the primary sites for lactylation. Therefore, site‐directed mutagenesis was employed to individually substitute lysine residues at positions 24, 197, 244, 249, 255, and 275 in USF2 with alanine. The USF2‐K255A‐ and USF2‐K275A‐mutated proteins in particular displayed significant reductions in lactylation (Figure 5D). The functional consequences of diminished lysine lactylation of USF2 on stimulation of *FABP4* promoter activity were assessed in dual‐luciferase reporter assays, which revealed that both USF2‐K255A and USF2‐K275A enhanced *FABP4* promoter activation compared to wild‐type USF2 (Figure 5E).

**Figure 5:**
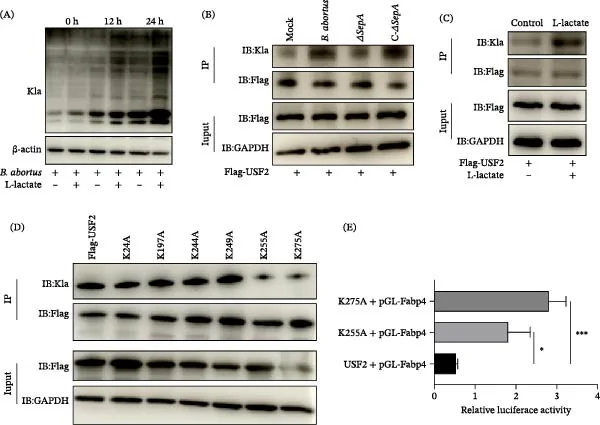
*B. abortus* modulates lactylation of transcription factor USF2. (A) Global lactylation levels in RAW264.7 macrophages during *B. abortus* infection (with or without exogenous L‐lactate treatment) were assessed by Western blotting at 0, 12, and 24 hpi using anti‐L‐lactyl lysine antibody. (B) RAW264.7 cells transfected with pCMV‐USF2‐3×Flag were infected with *B. abortus*, Δ*SepA* mutant, or *C*‐Δ*SepA* at 24 h post‐transfection. At 24 hpi, cell lysates were subjected to anti‐Flag Co‐IP, followed by Western blotting to detect lactylation of immunoprecipitated USF2. (C) RAW264.7 cells transfected with pCMV‐USF2‐3×Flag were treated with 20 mM L‐lactate prior to infection with *B. abortus*. USF2 lactylation was evaluated by anti‐Flag Co‐IP and Western blotting at 24 hpi and compared between the L‐lactate‐treated and untreated groups. (D) RAW264.7 cells were transfected with pCMV‐MCS‐3×Flag plasmids encoding wild‐type USF2 or the indicated lysine‐to‐alanine mutants (K24A, K197A, K244A, K249A, K255A, K275A). At 48 h post‐transfection, lactylation levels of immunoprecipitated Flag‐USF2 were determined by co‐IP and Western blotting. (E) HEK293T cells were cotransfected with pGL‐pro‐100 to −1, pRL‐CMV, and p3×FLAG‐CMV‐USF2, USF2‐K255A, or USF2‐K275A. Dual‐luciferase assays were performed 24 h post‐transfection to measure *FABP4* promoter activity and compare the USF2‐K255A and USF2‐K275A groups with the p3×FLAG‐CMV‐USF2 group. Data are presented as mean ± SD from three independent experiments (*n* = 3 per group). For comparisons between two groups, a two‐tailed unpaired Student’s *t*‐test was used. For comparisons among three or more groups, one‐way analysis of variance (ANOVA) followed by Dunnett’s post hoc test was performed to compare each experimental group with the control group.  ^∗^ *p*  < 0.05;  ^∗∗^ *p*  < 0.01;  ^∗∗∗^ *p*  < 0.001.

### 3.6. L‐Lactate May Modulate *B. abortus*‐Induced M1 Polarization of Macrophages via Suppression of FABP4

*Brucella* infection induces macrophage polarization toward the M1 phenotype. M1‐polarized macrophages produce excessive proinflammatory cytokines that are detrimental to the intracellular survival of the bacterium. However, lactate suppresses the production of proinflammatory mediators in macrophages [37]. We therefore hypothesized that L‐lactate may inhibit *B. abortus*‐mediated M1 polarization of macrophages. L‐lactate significantly downregulated the expression of IL‐1β, IL‐6, iNOS, TNF‐α, and CD86 in *B. abortus*‐infected RAW264.7 cells, which indicated the suppressive effect of L‐lactate on M1 polarization (Figure 6A–D,I,J). As L‐lactate mediates the inhibition of *FABP4* expression (Figure 3C), the contribution of FABP4 to this regulation was assessed. Knockdown of *FABP4* markedly reduced the expression of IL‐1β, IL‐6, iNOS, TNF‐α, and CD86 in *B. abortus*‐stimulated RAW264.7 cells (Figure 6E–H,K,L). Furthermore, neither treatment with L‐lactate nor *FABP4* knockdown induced significant changes in the mRNA expression levels of the M2 polarization‐associated markers TGF‐β and ARG1 in *B. abortus*‐infected RAW264.7 cells (Figure S4A–D). We also found that, compared with *B. abortus* and *C*‐Δ*SepA*, infection with Δ*SepA* significantly increased the expression of IL‐1β, IL‐6, and iNOS in RAW264.7 cells, though no significant difference was observed in TNF‐α expression (Figure S5A–D). Collectively, these results suggest that L‐lactate may attenuate *B. abortus*‐induced M1 polarization of macrophages, potentially through the suppression of FABP4 expression.

**Figure 6:**
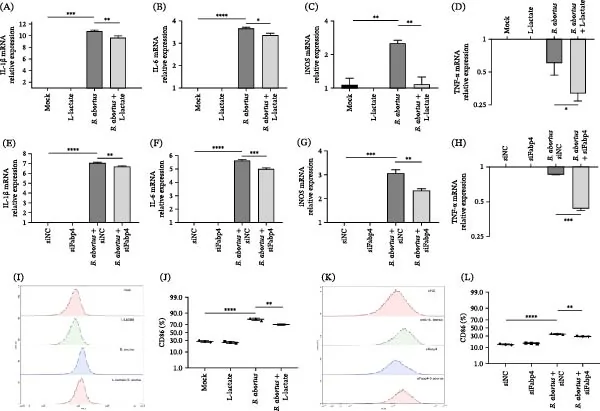
L‐lactate modulates *B. abortus*‐induced M1 polarization of RAW264.7 macrophages via suppression of FABP4. (A–D) The mRNA levels of IL‐1β, IL‐6, iNOS, and TNF‐α were quantified by qRT‐PCR in untreated or L‐lactate‐treated RAW264.7 cells with or without *B. abortus* infection. The *y*‐axis is presented on a log_2_ scale to visually emphasize symmetric changes in relative expression, with the *x*‐axis intersecting the *y*‐axis at 1 (reference value) to enhance clarity of deviations from the baseline. (E–H) The mRNA levels of IL‐1β, IL‐6, iNOS, and TNF‐α were quantified by qRT‐PCR in uninfected or *B. abortus*‐infected RAW264.7 cells transfected with siNC or siFabp4. The *y*‐axis is presented on a log_2_ scale to visually emphasize symmetric changes in relative expression, with the *x*‐axis intersecting the *y*‐axis at 1 (reference value) to enhance clarity of deviations from the baseline. (I, J) The CD86 protein levels were measured by flow cytometry in untreated or L‐lactate‐treated RAW264.7 cells with or without *B. abortus* infection. (K, L) The CD86 protein levels were analyzed via flow cytometry in uninfected or *B. abortus*‐infected RAW264.7 cells transfected with siNC or siFabp4. Experiments were repeated at least three times with similar results. Data are presented as mean ± SD from three independent experiments (*n* = 3 per group). For comparisons between two groups, a two‐tailed unpaired Student’s *t*‐test was used. For comparisons among three or more groups, one‐way analysis of variance (ANOVA) followed by Tukey’s HSD post hoc test for all pairwise comparisons was performed.  ^∗^ *p*  < 0.05;  ^∗∗^ *p*  < 0.01;  ^∗∗∗^ *p*  < 0.001;  ^∗∗∗∗^ *p*  < 0.0001.

### 3.7. L‐Lactate Inhibits *B. abortus*‐Induced Lipid Droplet Formation Through Regulation of FABP4 in Macrophages

FABP4 plays a critical role in lipid metabolism by regulating fatty acid transport and storage, thereby modulating lipid breakdown [38]. In view of the observation that L‐lactate suppresses FABP4 expression (Figure 3), we investigated whether L‐lactate influences lipid droplet formation through FABP4 regulation. Immunofluorescence results demonstrated that *B. abortus*, Δ*SepA*, and *C*‐Δ*SepA* groups all promote cellular lipid droplet formation. Notably, the Δ*SepA*‐infected group induced more extensive lipid droplet formation compared with the other infected groups (Figure 7A,D). Supplementation with L‐lactate significantly attenuated *B. abortus*‐mediated lipid droplet formation (Figure 7B,E). Furthermore, knockdown of *FABP4* also inhibited *B. abortus*‐induced lipid droplet formation (Figure 7C,F). In particular, L‐lactate reduced *B. abortus*‐triggered triglyceride (TG) accumulation in RAW264.7 cells (Figure 7G). In addition, *FABP4* knockdown substantially reduced cellular TG levels, whereas *FABP4* overexpression promoted TG production (Figure 7H,I). The functional relevance of lipid droplets in *B. abortus* intracellular proliferation was examined by treating RAW264.7 cells with oleic acid to induce lipid droplet biogenesis. Intriguingly, oleic acid treatment suppressed the intracellular proliferation of *B. abortus* at 24 hpi (Figure 7J). The same treatment with oleic acid had no significant effect on the intracellular survival of *B. abortus* in RAW264.7 cells at 2 hpi (Figure 7K). These findings collectively establish a regulatory axis involving L‐lactate, Fabp4, and lipid metabolism in the modulation of bacterial intracellular survival.

**Figure 7:**
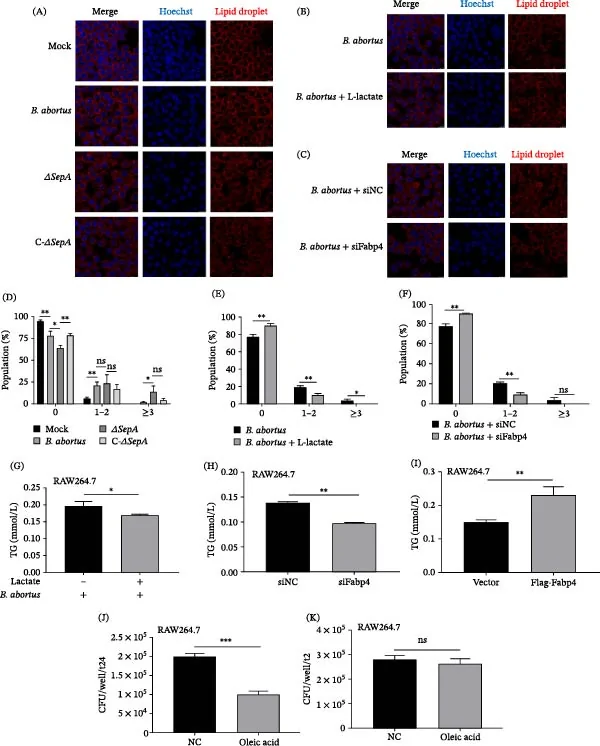
L‐lactate inhibits *B. abortus*‐induced lipid droplet formation through regulation of FABP4 in RAW264.7 macrophages. (A) Lipid droplets in RAW264.7 cells were visualized by immunofluorescence at 24 hpi with *B. abortus*, Δ*SepA* mutant, *C*‐Δ*SepA* complemented strain, or in uninfected controls. (B) Lipid droplets were visualized in RAW264.7 cells treated with or without L‐lactate and infected or uninfected with *B. abortus* at 24 hpi. (C) RAW264.7 cells transfected with siNC or siFabp4 were infected with *B. abortus*, and lipid droplets were detected by immunofluorescence at 24 hpi. (D–F) From the fluorescence images (A–C), the frequencies of lipid droplets were calculated by counting the populations of cells containing 0, 1–2, or ≥3 lipid droplets (total number of cells = 150–250 for each experiment) and plotted as bar graphs. (G) Triglyceride (TG) levels in *B. abortus*‐infected RAW264.7 cells treated with or without L‐lactate at 24 h posttreatment with L‐lactate. (H) TG levels in *B. abortus*‐infected RAW264.7 cells transfected with siNC or siFabp4 at 24 hpi. (I) TG levels in *B. abortus*‐infected RAW264.7 cells were transfected with p3×FLAG‐CMV empty vector or p3×FLAG‐CMV‐Fabp4 at 24 hpi. (J, K) Intracellular bacterial loads in *B. abortus*‐infected RAW264.7 cells treated with or without oleic acid were quantified at 24 hpi (J) and 2 hpi (K). Experiments were repeated at least three times with similar results. Data are presented as mean ± SD from three independent experiments (*n* = 3 per group). For comparisons between two groups, a two‐tailed unpaired Student’s *t*‐test was used. For comparisons among three or more groups, one‐way analysis of variance (ANOVA) followed by Tukey’s HSD post hoc test for all pairwise comparisons was performed.  ^∗^ *p*  < 0.05;  ^∗∗^ *p*  < 0.01;  ^∗∗∗^ *p*  < 0.001.

### 3.8. USF2 Lysine Lactylation Modification Regulates M1 Polarization of Macrophages and Lipid Droplet Formation

Collectively, the above experimental findings demonstrate that USF2 lysine lactylation modification regulates the expression of Fabp4. Given that Fabp4 has been well established to participate in macrophage polarization and lipid droplet biogenesis, we therefore postulate that USF2 lactylation may also exert a regulatory effect on these two biological processes in macrophages. Consistent with our hypothesis, overexpression of wild‐type USF2 significantly upregulated the mRNA levels of M1 macrophage signature proinflammatory cytokines, including IL‐1β, IL‐6, iNOS, and TNF‐α, in RAW264.7 cells. This promotive effect was further potentiated when the lactylation‐deficient point mutants USF2‐K255A or USF2‐K275A were overexpressed (Figure 8A–D). In addition, USF2 overexpression facilitated lipid droplet formation in RAW264.7 cells, and this stimulatory effect was more pronounced in cells transfected with USF2‐K255A or USF2‐K275A mutants (Figure 8E,F). We further demonstrated that overexpression of wild‐type USF2, USF2‐K255A, or USF2‐K275A markedly suppressed the intracellular proliferation of *B. abortus* at 24 hpi; the intracellular survival of *B. abortus* remained unaffected at 2 hpi (Figure 8G,H). Moreover, relative to wild‐type USF2, the USF2‐K255A and USF2‐K275A mutants exerted a more potent inhibitory effect on the intracellular replication of *B. abortus* (Figure 8G).

**Figure 8:**
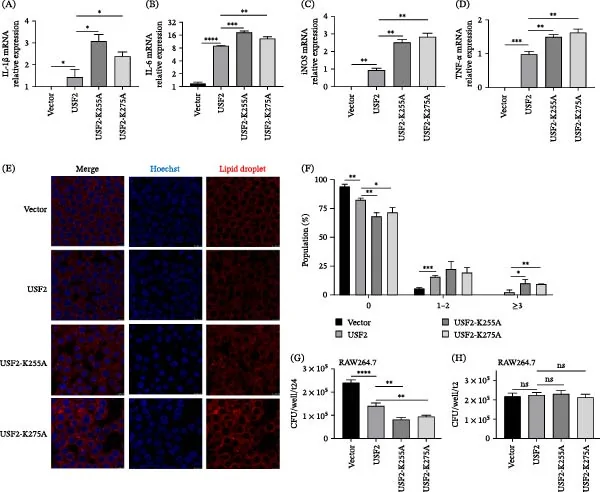
Effects of USF2 on cytokines, lipid droplets, and intracellular proliferation of *B. abortus* in RAW264.7 cells. (A–D) RAW264.7 cells were transfected with p3×FLAG‐CMV, p3×Flag‐USF2, or lysine‐to‐alanine (K‐to‐A) mutants (p3×Flag‐USF2‐K255A or p3×Flag‐USF2‐K275A). At 24 h post‐transfection, mRNA levels of IL‐1β, IL‐6, iNOS, and TNF‐α were determined by qRT‐PCR. (E) RAW264.7 cells were transfected with p3×FLAG‐CMV, p3×Flag‐USF2, p3×Flag‐USF2‐K255A, or p3×Flag‐USF2‐K275A. Lipid droplets in RAW264.7 cells were visualized at 48 h post‐transfection. (F) From the fluorescence images (E), the frequencies of lipid droplets were calculated by counting the populations of cells containing 0, 1–2, or ≥3 lipid droplets (total number of cells = 150–250 for each experiment) and plotted as bar graphs. (G, H) RAW264.7 cells were transfected with p3×FLAG‐CMV, p3×Flag‐USF2, p3×Flag‐USF2‐K255A, or p3×Flag‐USF2‐K275A. At 24 h post‐transfection, cells were infected with *B. abortus* at MOI of 200. Intracellular bacterial CFU counts were determined at 24 hpi (G) and 2 hpi (H). Experiments were repeated at least three times with consistent results. Data are presented as mean ± SD from three independent experiments (*n* = 3 per group). For comparisons between two groups, a two‐tailed unpaired Student’s *t*‐test was used. For comparisons among three or more groups, one‐way analysis of variance (ANOVA) followed by Tukey’s HSD post hoc test for all pairwise comparisons was performed.  ^∗^ *p*  < 0.05;  ^∗∗^ *p*  < 0.01;  ^∗∗∗^ *p*  < 0.001;  ^∗∗∗∗^ *p*  < 0.0001.

## 4. Discussion

Previous analyses [4, 8] and our current findings conclusively demonstrate that *Brucella* infection enhances lactate production in macrophages. However, the role of lactate in the intracellular survival of the bacterium remains unexplored. Nevertheless, emerging evidence suggests that the molecule plays critical roles in the intracellular persistence of pathogenic microorganisms [20–25], which prompted us to investigate the functional significance of lactate during *Brucella* infection of host cells. *B. abortus* infection of macrophages markedly upregulated L‐lactate production (Figure 1A). Intriguingly, elevation of cellular lactylation levels following *B. abortus* infection was also observed, which may be attributed to increased L‐lactate production (Figure 1B). As lactate represents the terminal product of glycolysis, its cellular levels are directly influenced by glycolytic activity. The impact of lactate on *Brucella* intracellular proliferation was elucidated by modulating intracellular L‐lactate concentrations through exogenous supplementation with L‐lactate or glycolytic inhibition with 2‐DG. This strategy revealed that L‐lactate positively regulates the intracellular proliferation of *B. abortus* by a mechanism that affects host cells rather than directly impacting the bacterium (Figure 1C–F). These findings suggest that *Brucella* may hijack host glycolytic pathways to stimulate L‐lactate production, with subsequent exploitation of L‐lactate‐mediated modification of host cellular processes to regulate intracellular proliferation.

Comparative proteomic analysis of cells with and without *B. abortus* infection was conducted to elucidate the mechanism by which the bacterium affects L‐lactate production in macrophages. Slc2a1 was among the differentially expressed proteins identified. Slc2a1 is a glycolysis‐related factor that is critical for glucose transport and plays a pivotal role in the glycolytic pathway [39, 40]. Our findings demonstrate that Slc2a1 positively regulates intracellular proliferation of *B. abortus* and cellular lactylation levels in macrophages, thus exhibiting a similar effect as L‐lactate (Figure 2). The type‐IV secretion system (T4SS) is a key virulence factor that is essential for the intracellular survival of *Brucella* [41]. The system is a 12‐protein complex encoded by the *virB* operon in *Brucella*. The complex acts through 15 secreted effector proteins, which impinge on vital host signaling pathways, thereby helping the bacterium to survive and replicate in the host [42]. We speculated that the T4SS in *B. abortus* may play an important role in promoting the expression of the host Slc2a1 protein. Indeed, overexpression of the T4SS effector protein SepA and analysis of the *B. abortus* sepA deletion strain revealed that SepA positively regulates Slc2a1 expression and host cell lactylation levels (Figure 2I–L). Stimulation of Slc2a1 expression by SepA accords with the finding that SepA is involved in regulating *Brucella* survival within host cells [43]. The observation that *Brucella* modulates host cell metabolic activity via the T4SS emphasizes the key role that metabolic alterations play in bacterial intracellular survival, in this case, via the effector protein SepA exerting a pivotal function in regulating host glycolysis. The molecular mechanism that underpins SepA‐mediated regulation of Slc2a1 expression remains to be elucidated, and further investigation to uncover this mechanism has been initiated.

*B. abortus* infection markedly upregulated FABP4 expression in macrophages, whereas L‐lactate treatment suppressed its expression. Furthermore, functional studies demonstrated that FABP4 negatively regulates *B. abortus* intracellular proliferation (Figure 3). Thus, L‐lactate facilitates bacterial multiplication by inhibiting FABP4 expression, which raises a critical mechanistic question: through what molecular pathway does L‐lactate mediate *FABP4* transcriptional regulation? Lactate exerts a crucial regulatory role in histone modification via histone lactylation modification [26]. However, lactylation modification is not confined to histones but also encompasses nonhistone functional proteins, thereby expanding the epigenetic and posttranslational regulatory significance of the process [28–33]. We hypothesized that L‐lactate suppresses FABP4 expression by modulating lactylation‐dependent transcriptional regulation. Initial findings that L‐lactate significantly inhibited *FABP4* promoter activity aligned with this hypothesis. Subsequently, USF2 was identified as a transcriptional regulator of *FABP4* (Figure 4). Moreover, USF2 is subject to lactylation modification, with lysine residues at positions 255 and 275 serving as lactylation sites. Mutation of these residues resulted in a substantial enhancement of USF2‐mediated activation of the *FABP4* promoter (Figure 5). Thus, L‐lactate may suppress FABP4 expression through lysine lactylation of USF2. Intriguingly, we also discovered that USF2 undergoes lysine lactylation during uninfected cell growth conditions, which may be attributable to baseline L‐lactate production inherent to cellular metabolism (Figure 5B,C). Although this endogenous L‐lactate is sufficient to induce lysine lactylation of USF2, *B. abortus* infection or exogenous L‐lactate supplementation amplifies intracellular L‐lactate accumulation, thereby enhancing USF2 lysine lactylation. Thus, escalating L‐lactate levels within physiologically tolerable thresholds may progressively intensify USF2 lysine lactylation and lead to incremental suppression of *FABP4* expression. Macrophage cells may upregulate *FABP4* expression via an unidentified mechanism to facilitate *B. abortus* clearance. Intracellular *B. abortus* counteracts this host defense strategy by enhancing L‐lactate production in host cells to modulate USF2 lysine lactylation, which subsequently suppresses *FABP4* expression. This dynamic host‐pathogen interplay may result in partial bacterial elimination while enabling *Brucella* to achieve optimal survival within the host microenvironment. One limitation of this study is that the direct effect of USF2 lysine lactylation on DNA binding was not verified by in vitro biochemical assays such as EMSA. Further studies are needed to provide direct biophysical evidence for this regulatory mechanism.

*B. abortus* infection induces macrophage polarization toward an M1 phenotype, whereas *B. abortus* preferentially survives within M2‐polarized macrophages [44–46]. L‐lactate plays a pivotal role in regulating macrophage polarization, particularly by promoting the M2 phenotype [47, 48]. We hypothesized that L‐lactate may enhance the intracellular proliferation of *B. abortus* by modulating macrophage polarization. Indeed, L‐lactate significantly suppressed *B. abortus*‐induced M1 polarization of RAW264.7 cells, which suggests that the molecule influences the intracellular survival of *B. abortus* by modulating macrophage polarization. As we observed that L‐lactate promotes *B. abortus* intracellular proliferation by suppressing FABP4 expression, we further demonstrated that FABP4 may upregulate *B. abortus*‐induced M1 polarization in macrophages, which indicates that L‐lactate regulates M1 polarization through Fabp4 inhibition (Figure 6). As FABP4 plays a critical role in lipid metabolism [38], we investigated whether L‐lactate modulates macrophage lipid metabolism via FABP4 suppression. Consistent with this hypothesis, L‐lactate inhibited lipid droplet formation and TG synthesis in macrophages by regulating FABP4, thereby promoting *B. abortus* intracellular proliferation (Figure 7). These results underscore the pivotal roles of L‐lactate and FABP4 in regulating *B. abortus* intracellular proliferation, not only by influencing macrophage polarization but also by altering cellular lipid metabolism. As macrophage polarization and lipid metabolism are intricately linked to host immunity [49, 50], elucidating the interplay between *B. abortus* and these processes is crucial for developing strategies to prevent and control brucellosis. Thus, further investigation will aim to unravel the molecular mechanisms through which L‐lactate and FABP4 regulate host cell polarization and lipid metabolism during *B. abortus* infection.

Although subsequent reports have demonstrated that *Brucella* infection of distinct cell types substantially upregulates cellular glycolytic activity and that alterations in glycolytic flux affect *Brucella* intracellular survival [4–8], our study further delineates how glycolysis regulates *Brucella* intracellular survival—namely, the glycolytic product L‐lactate participates in regulating bacterial intracellular survival in RAW264.7 cells via lysine lactylation. In this process, the effector protein SepA plays a critical role. Similarly, in *Brucella*‐mediated regulation of host‐cell NAD metabolism, the effector proteins BtpA and BtpB also serve pivotal functions [9]. Collectively, these findings indicate that *Brucella* effector proteins exert important functions in modulating host cell metabolism.

In natural hosts, the metabolic adaptation of *Brucella* displays characteristics markedly distinct from those observed in macrophage infection models. Ruminant placental trophoblasts are rich in erythritol, and *B. abortus* and *B. melitensis* preferentially utilize erythritol rather than glucose as an energy source; this substrate preference is considered a key metabolic basis for massive bacterial proliferation in the placenta and subsequent abortion. Comparative genomics further reveals that zoonotic *Brucella* species (including *B. abortus* and *B. melitensis*) have convergently lost the Entner–Doudoroff pathway in hexose catabolism and instead rely obligately on the pentose phosphate pathway (PPP); this metabolic trait is associated with virulence phenotypes in mice and has been interpreted as a metabolic evolutionary imprint of adaptation to livestock hosts [51]. This implies that within the reproductive tract niche of natural hosts, the carbon metabolism strategy of *Brucella* centers on PPP and erythritol utilization, whereas the role of glycolytic flux may be far less prominent than in macrophages. Whether our lactylation mechanism operates within the placental trophoblast, a natural niche, remains to be validated; however, it is plausible that during the macrophage‐rich chronic infection stage or acute bacteremia, Warburg‐like glycolytic reprogramming and subsequent lactylation modifications are more likely to constitute a meaningful regulatory layer.

In conclusion, our findings reveal a dual regulatory network in *B. abortus* that exploits host lactate metabolic reprogramming via the SepA/Slc2a1 and L‐lactate/USF2/FABP4 axes to establish an intracellular infection niche. These findings may provide novel insights into the prevention and control of brucellosis and suggest new therapeutic approaches to combat the disease (Figure 9).

**Figure 9:**
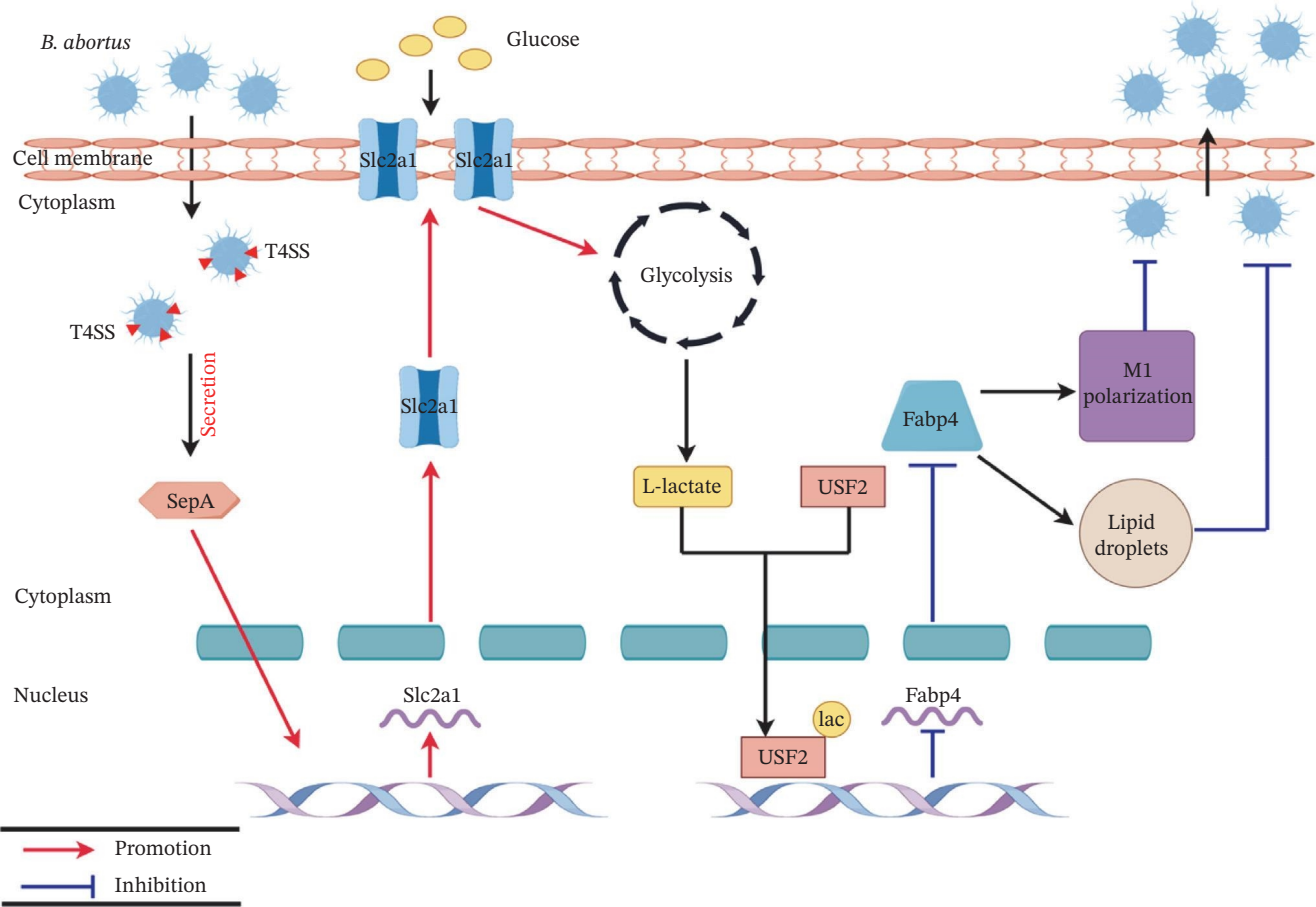
Schematic diagram of the mechanism underlying L‐lactate‐promoted intracellular proliferation of *Brucella abortus*. Following infection of *B. abortus* in RAW264.7 cells, the T4SS secretes the effector protein SepA, which upregulates the expression of the host cell Slc2a1. This enhances glycolytic activity, leading to increased synthesis of L‐lactate. The elevated L‐lactate exacerbates lysine lactylation modification of the upstream transcription factor USF2, which subsequently suppresses the expression of FABP4. Consequently, FABP4‐mediated M1 polarization and lipid droplet formation are attenuated, thereby creating a favorable intracellular niche that promotes *B. abortus* proliferation. This image was drawn using Figdraw.

Despite these findings, this study has several limitations. First, all experiments were performed using the RAW264.7 macrophage‐like cell line. Although RAW264.7 cells are widely used to study *Brucella* infection, they are not the natural host of *Brucella* and cannot fully reproduce the complex in vivo environment, including tissue‐specific immune responses, cell–cell interactions, and systemic inflammatory networks. Second, we did not validate the virulence of the SepA mutant or the expression changes of FABP4 in mouse infection models. Therefore, the in vivo roles of SepA and FABP4 in *Brucella* pathogenesis remain to be determined. Future studies using BMDMs and mouse infection models will be necessary to confirm our in vitro findings and to elucidate the in vivo relevance of the SepA–FABP4 axis. Until then, the conclusions of this study should be interpreted within the context of an in vitro macrophage infection model.

## Author Contributions

Gaowa Wudong and Xiaofang Liu conceptualized and designed the experiments. Ye Yuan, Yunyi Zhai, Xin Wang, and Yaping Zu analyzed the data. Wei Liu, Dong Zhou, Aihua Wang, and Yaping Jin contributed to the revision of critical content in the manuscript. Yuanhao Yang designed and conducted experiments and wrote the manuscript with input from all the authors.

## Funding

This work was supported by the Key Research and Development Program of Ningxia Hui Autonomous Region (Grant 2024BBF01005) and the National Natural Science Foundation of China (Grant 32373016).

## Ethics Statement

The authors have nothing to report.

## Consent

The authors have nothing to report.

## Conflicts of Interest

The authors declare no conflicts of interest.

## Supporting Information

Additional supporting information can be found online in the Supporting Information section.

## Supporting information


**Supporting Information** Figures S1–S6 are provided in the Supporting Information. Figure S1: Effects of L‐lactate and 2‐DG on intracellular survival of *B. abortus* A19 and the impact of Slc2a1 knockdown on *B. abortus*‐induced L‐lactate production in host cells. Figure S2: Effect of Slc2a1 on the intracellular survival of *B. abortus* A19. Figure S3: Fabp4 regulates intracellular survival of *B. abortus* A19 in RAW264.7 cells. Figure S4: L‐lactate and Fabp4 do not affect ARG1 and TNF‐β mRNA levels in RAW264.7 cells. Figure S5: Effects of *B. abortus* A19 ΔSepA deletion mutant on cytokine expression. Figure S6: The transcription factor Vezf1 modulates the promoter activity of Slc2a1.

## Data Availability

The mass spectrometry proteomics data have been deposited to the ProteomeXchange Consortium via the PRIDE partner repository with the dataset identifier PXD063435. The data within the database have been made publicly accessible.
