# Supplementary material for: Macrophage USF2 Lactylation‐Mediated FABP4 Suppression Promotes Intracellular Proliferation of Brucella
Source: Transbound Emerg Dis. 2026 Nov 4;2026:3930677. doi: 10.1155/tbed/3930677 (PMC13736068; doi:10.1155/tbed/3930677)
Supplement: Supplementary file 1 [file TBED-2026-3930677-s001.docx]

**Figure S1**

**Figure S1** Effects of L-lactate and 2-DG on intracellular survival of *B. abortus* A19 and the impact of Slc2a1 knockdown on *B. abortus*-induced L-lactate production in host cells. (A) RAW264.7 cells infected with *B. abortus* A19 were treated with 0, 10, or 20 mM L-lactate. Intracellular *B. abortus* A19 burden was quantified via CFU assay at 2 h post-treatment. (B) RAW264.7 cells infected with *B. abortus* A19 were treated with 0, 0.5, or 2 mM 2-deoxy-D-glucose (2-DG). Intracellular bacterial load was determined using CFU assay at 2 h post-treatment. (C) RAW264.7 cells were transfected with siNC or siSlc2a1-1470 (50 nM) and infected with *B. abortus* A19 (MOI=200) after 24 h; intracellular L-lactate levels in RAW264.7 cells were then measured. Experiments were repeated at least three times with similar results. **P<0.01.

**Figure S2**

**Figure S2** Effect of Slc2a1 on the intracellular survival of *B. abortus* A19. (A) RAW264.7 cells were transfected with p3xFLAG-CMV or p3xFLAG-CMV-Slc2a1, followed by infection with *B. abortus* A19 at MOI of 200 after 24 h. Cells were harvested an additional 2 h post-infection, and intracellular bacterial CFU counts were determined. (B) RAW264.7 cells were transfected with siNC or siSlc2a1-1470 (50 nM), after24h, infected with *B. abortus* A19 (MOI=200) at 2 h post-transfection. Cells were harvested 2 h post-infection, and intracellular bacterial CFU counts were determined.

**Figure S3**

**Figure S3** Fabp4 regulates intracellular survival of *B. abortus* A19 in RAW264.7 cells. (A, B) RAW264.7 cells were transfected with p3xFLAG-CMV or p3xFLAG-CMV-Fabp4, then infected with *B. abortus* A19 *sepA* deletion mutant (*ΔSepA*), or its complemented strain (*C-ΔSepA*). Intracellular bacterial loads were quantified at 24 h post-infection. (C, D) RAW264.7 cells were transfected with siNC or siFabp4-432, followed by infection with *ΔSepA*, or *C-ΔSepA* at 24 h post-transfection. Intracellular bacterial loads were analyzed at 24 h post-infection. Experiments were repeated at least three times with consistent results. **P<0.01; ***P<0.001.

**Figure S4**

**Figure S4** L-lactate and Fabp4 do not affect ARG1 and TNF-β mRNA levels in RAW264.7 cells. (A, B) The mRNA levels of ARG1 and TNF-β were detected by qRT-PCR in untreated or L-lactate-treated RAW264.7 cells, either uninfected or infected with *B. abortus* A19. (C, D) The mRNA levels of ARG1 and TNF-β were detected via qRT-PCR in uninfected or *B. abortus* A19-infected RAW264.7 cells transfected with siNC or siFabp4.

**Figure S5**

**Figure S5** Effects of *B. abortus* A19 Δ*SepA* deletion mutant on cytokine expression. (A-D) The mRNA levels of IL-1β, IL-6, iNOS, and TNFα were detected by qRT-PCR in RAW264.7 cells either uninfected or infected with *B. abortus* A19 wild-type, sepA deletion mutant (*ΔSepA*), or its complemented strain (*C-ΔSepA*). The y-axis is presented on a Log2 scale to visually emphasize symmetric changes in relative expression, with the x-axis intersecting the y-axis at 1 (reference value) to enhance clarity of deviations from the baseline. Experiments were repeated at least three times with consistent results. **P<0.01; ***P<0.001.

**Figure S6**

**Figure S6** The transcription factor Vezf1 modulates the promoter activity of *Slc2a1*. (**A**) HEK293T cells were co-transfected with pRL-CMV (Renilla control) and pGL4.10-based reporter plasmids containing the indicated *Slc2a1* promoter fragments (pGL-pro-2000 to -1). Dual-luciferase assays were performed 24 h post-transfection to assess promoter activity. (B) HEK293T cells were co-transfected with pGL-pro-2000 to -1, and either p3×FLAG-CMV or p3×FLAG-CMV-SepA. *Slc2a1* promoter activation was measured 24 h after transfection and compared among the p3×FLAG-CMV and p3×FLAG-CMV-SepA. (**C**) HEK293T cells were co-transfected with pRL-CMV and pGL4.10-based reporter plasmids containing the indicated *Slc2a1* promoter fragments (pGL-pro-2000 to -1, -1500 to -1, -1000 to -1, or -500 to -1). Dual-luciferase assays were performed 24 h post-transfection to assess promoter activity. (**D**) HEK293T cells were co-transfected with pRL-CMV and pGL4.10-based reporter plasmids containing the indicated *Slc2a1* promoter fragments (pGL-pro-500 to -300, -300 to -1 or -150 to -1). Dual-luciferase assays were performed 24 h post-transfection to assess promoter activity. (**E, F**) Computational prediction of transcription factor binding sites in the -150 to -1 region of the *Slc2a1* promoter using AnimalTFDB v4.0. (**G**) HEK293T cells were co-transfected with pRL-CMV and wild-type or mutant pGL-pro-150 to -1 constructs (Vezf1 mut), and promoter activity was compared via dual-luciferase assays. (**H**) HEK293T cells were co-transfected with pRL-CMV, pGL-pro-2000 to -1, and either pCMV-3×Flag or pCMV-Vezf1-3×Flag. *Slc2a1* promoter activation was measured 24 h after transfection and compared among the pCMV-3×Flag and pCMV-Vezf1-3×Flag. (I) RAW264.7 cells were infected with *B. abortus*, the *ΔSepA* mutant, or *C-ΔSepA* at 24 h post-transfection. Total RNA was subsequently extracted, and Vezf1 mRNA levels were quantified by RT-qPCR; furthermore, comparative analysis of Vezf1 mRNA expression was conducted across the different infection groups. Experiments were repeated at least three times with consistent results. **P<0.01; ***P<0.001; ****P<0.0001.
